# Contexts of vulnerability and the acceptability of new biomedical HIV prevention technologies among key populations in South Africa: A qualitative study

**DOI:** 10.1371/journal.pone.0191251

**Published:** 2018-02-08

**Authors:** Millicent Atujuna, Peter A. Newman, Melissa Wallace, Megan Eluhu, Clara Rubincam, Ben Brown, Linda-Gail Bekker

**Affiliations:** 1 Desmond Tutu HIV Foundation, Health Sciences Faculty, Institute of Infectious Disease, University of Cape Town, Cape Town, South Africa; 2 Factor-Inwentash Faculty of Social Work, University of Toronto, Toronto, Ontario, Canada; University of New South Wales, AUSTRALIA

## Abstract

**Background:**

New biomedical prevention technologies (NPTs) may contribute to substantially reducing incident HIV infections globally. We explored acceptability and preferences for NPTs among key and other vulnerable populations in two South African townships.

**Methods:**

We conducted six focus groups and 12 in-depth interviews with adolescents, and adult heterosexual men, women, and men who have sex with men (MSM) (n = 48), and eight in-depth interviews with key informant healthcare workers. The interview guide described pre-exposure prophylaxis (PrEP), vaginal rings, rectal microbicides and HIV vaccines, and explored acceptability and product preferences. Focus groups and in-depth interviews (45–80 minutes) were conducted in Xhosa, audiotaped, and transcribed and translated into English. Data were coded and reviewed using framework analysis with NVivo software.

**Results:**

Overall, initial enthusiasm and willingness to use NPTs evolved into concerns about how particular NPTs might affect or require alterations in one’s everyday lifestyle and practices. Different product preferences and motivations emerged by population based on similarity to existing practices and contexts of vulnerability. Adult women and female adolescents preferred a vaginal ring and HIV vaccine, motivated by longer duration of protection to mitigate feared repercussions from male partners, including threats to their marriage and safety, and a context of ubiquitous rape. Male adolescents preferred an HIV vaccine, seen as protection in serodiscordant relationships and convenient in obviating the HIV stigma and cost involved in buying condoms. Adult men preferred PrEP, given familiarity with oral medications and mistrust of injections, seen as enabling serodiscordant couples to have a child. MSM preferred a rectal microbicide given familiarity with gel-based lubricants, with concerns about duration of protection in the context of unplanned consensual sex and rape.

**Conclusions:**

Biomedical interventions to prevent HIV transmission, rather than obviating social-structural factors that produce vulnerability, may be limited by these same factors. Implementation of NPTs should engage local communities to understand real-world constraints and strategise to deliver effective, multi-level combination prevention.

## Introduction

New biomedical prevention technologies (NPTs) may contribute to substantially reducing incident HIV infections globally. Oral pre-exposure prophylaxis (PrEP) has been shown to be highly effective in clinical trials across diverse populations [1–4], though it is currently licensed in few countries. Recent large-scale trials of a vaginal ring containing an antiretroviral drug have demonstrated safety and efficacy among adult women [5,6], with trials in progress among adolescents and regulatory approvals underway. Initial rectal microbicide trials demonstrate safety and acceptability among men who have sex with men (MSM), and women [7,8]. New HIV vaccine trials currently underway aim to build on the partial efficacy of the investigational RV144 vaccine [9,10]. Thus, NPTs are in various stages of progress in the HIV prevention pipeline.

Impetus for the development of NPTs stems in part from growing acknowledgment of the limitations of behavioural interventions alone in controlling an epidemic undergirded by social-structural factors [11–13]. The constraints of behaviourally-mediated prevention are particularly evident among individuals in economically, socially, politically and culturally disadvantaged positions who face limitations in their ability to make and enforce decisions around safer sex practices. NPTs such as PrEP, vaginal rings, rectal microbicides, and HIV vaccines hold out the opportunity for protection against HIV that is not necessarily contingent on negotiation with sexual partners in each sexual encounter, as is the case with the predominant existing technology, the male condom. NPTs may be particularly valuable in contexts such as South Africa, with a generalised epidemic and an array of social-structural factors associated with HIV risk behaviours [14].

A nexus of social-structural factors undergirds the epidemiology of HIV in South Africa through past and ongoing political, social and economic changes as the country transitioned from colonial, post-colonial and Apartheid eras. Importantly, these changes have impacted on the political economy of sex and sexual relationships, with marriage in decline and men’s and women’s movement across the country on the rise [15,16]. Together, these factors provide favourable conditions for partner concurrency as well as transactional sex, both of which are associated with higher risk of HIV infection.

Such complex challenges have made it particularly appealing to consider mass provision of NPTs to key (i.e., at increased risk for HIV infection across contexts, such as MSM) and other vulnerable populations (i.e., particularly vulnerable to HIV infection in certain contexts, such as adolescents), as defined by WHO [17]. Widespread implementation of NPTs in these populations may be seen to provide protection that is less encumbered by social-structural barriers—for example, by obviating the need for sexual negotiation at each sexual encounter as in the case of male condoms. However, in practice, clinical trials of NPTs have demonstrated adherence-related challenges that have substantially compromised product efficacy, as in vaginal and oral PREP trials in South Africa [18–21]. These trials support the need for greater attention to the complex realities of people’s everyday lives, and the impact of social-structural contexts on location-population-specific product acceptability and preferences [22–24]. NPT preparedness and implementation science studies are needed to explore product acceptability and evaluate interventions in real-world settings in order to facilitate the translation of technological advances into everyday use [25–27]. Such investigations are desirable well before NPT products are ready for dissemination, to promote the most comprehensive understanding of the “preconditions for their demand, acceptance and use by communities” [28].

Several studies across key and vulnerable populations depict high acceptability of various NPTs with different preferences by population [24,29,30]. The majority of NPT acceptability studies in South Africa have understandably focused on adult women, with a need for additional attention to adolescents, MSM and heterosexual men [31–33]. To address these gaps in research, we explored acceptability and preferences for NPTs, and factors associated with acceptability, across multiple populations in South African townships.

## Materials and methods

We used a qualitative approach to enable in-depth exploration of the perspectives and lived experience of diverse participants from peri-urban communities at heightened vulnerability to HIV. This study is reported in accordance with the COREQ (consolidated criteria for reporting qualitative research) guidelines (see S1 Appendix).

### Setting

Individuals were recruited from key and vulnerable populations in two informal peri-urban communities near Cape Town, South Africa, including MSM, adolescents and heterosexual adults [17]. These communities emerged as a product of the apartheid history of creating locations for black people, and grew in response to people moving in large numbers from impoverished conditions in their provinces of origin to Cape Town in search of employment. Generally, people maintain contact with and visit their families in their areas of origin. The populations of these communities are maintained by ongoing internal movement of people looking for jobs, some of whom may have relatives in these areas already. The living conditions and infrastructure are poor in these informal communities, characterised by high levels of poverty, overcrowded housing, unemployment, crime, alcohol and drug use, and sexual violence [34]. Both participating communities suffer from a generalised HIV epidemic, with high HIV prevalence documented among adult heterosexual men and women (25.0% [34]), MSM (25.5% [35]) and adolescents (7.1–8.5% nationally [36]). While these factors influenced our choice of study settings, the two sites were also selected because members of these communities had participated in HIV prevention clinical trials conducted in partnership with many multinational networks, including PrEP [4,37], vaginal microbicide [38] and HIV vaccine trials [39].

### Participants and data collection

Participants were purposively sampled with the aim of recruiting adolescents, MSM and heterosexual adults, some of whom had prior experience with clinical trials relating to NPTs and others who did not. As our aim, apropos of a qualitative approach, was not to generalise but to delve into perspectives and experiences of different key and vulnerable populations, we conducted two focus group discussions (FGDs) and four in-depth interviews (IDIs) within each population to enable exploration of possible commonalities and differences across populations. By design, we conducted separate FGDs (and IDIs) by participant gender and sexual orientation to facilitate exploration of possible differences along these lines. Though our original aim was also to compare and contrast responses from trial-experienced and trial-naïve participants, in practice, participants were reluctant to identify themselves as having prior experience with trials; thus our sample was dominated by those identifying themselves as ‘trial-naïve’. Trial-experienced participants were recruited from the existing trial databases of the Desmond Tutu HIV Foundation (DTHF); those who met inclusion criteria of age and being HIV-negative (at study exit or currently HIV-negative and still in a trial) were contacted by telephone and invited to participate. Those who indicated interest after the study was explained were scheduled for IDIs or FGDs according to their preferences. For trial-naïve participants, the DTHF recruitment team visited the communities and provided information to individuals about the study. Those who indicated interest and met the criteria for inclusion were scheduled for interviews or focus groups.

Focus groups were conducted in community offices of DTHF or in the professional offices of healthcare providers (HCP). During the IDIs and FGDs, no other personnel outside of the study team were present. For IDIs, only the interviewee and interviewer were present; for FGDs, a note taker and a facilitator were present along with the participants. No interviews were done in public settings, both to enable privacy and to ensure better quality of the recording. We conducted eight key informant interviews (KIIs) with local HCP, community outreach workers, HIV counsellors, and policy experts to elicit further context for understanding possible use of NPTs in the future by their clients.

Six DTHF staff members, three men and three women, conducted the IDIs and FGDs. The key informants were interviewed in English by a trained social behavioural scientist with a masters degree. Other participants were interviewed in either Xhosa or English or a combination of the two, as dictated by the comfort and preferences of participants. MSM IDIs and FGDs were conducted by staff members who shared the same sexual identity as participants to encourage comfort and candour. Interviews and FGDs with adolescents, heterosexual men, and heterosexual women were conducted by two female staff members who had both received training in qualitative interviewing techniques.

Prior to the IDIs and FGDs, interviewers and facilitators followed a rigorous process of informed consent. At the beginning of FGDs and IDIs, the facilitator/interviewer delivered a brief script on NPTs: vaginal microbicides (both ring and gel), rectal microbicides, HIV vaccines, and PrEP. The script included a lay language description of each product, estimated efficacy levels based on previous clinical trials, and the fact that none of these products was available in South Africa (PrEP was approved in South Africa in November 2015). Participants were shown a vaginal ring, a PrEP pill, and pictures of an HIV vaccine injection, and vaginal and rectal microbicide gels. The topic guides included questions and probes that addressed general awareness of NPTs; acceptability and possible use of NPTs; views on dosing regimens/frequency and distribution sites; perceived risk of HIV infection and current methods of protection; and understanding of product efficacy (see S2 and S3 Appendices). The key informant interview guide mirrored the topic guides for FGDs and IDIs, and elicited perspectives on their clients and the communities in which they worked (see S4 Appendix). FGDs and IDIs lasted from 45–80 minutes and were audio-taped. Participants were given a transportation reimbursement voucher worth 50 Rand ($5 US). Two trained bilingual Xhosa speaking personnel conducted FGDs and IDIs, and later transcribed them into Xhosa before translating them into English. Each Xhosa transcription and English transcript was cross-checked by the other transcriber to ensure quality. The lead author on the study (MA) then read the whole English transcript to identify any remaining errors; these were sent back to the transcription team for correction.

This study received approval from the Research Ethics Board of the University of Toronto and the Health Science Faculty Research Ethics Committee (HREC) of the University of Cape Town. All participants provided written informed consent and adolescent participants (<18 years-old) received written parental/caregiver consent before participating in the study. All consent forms were approved by the UCT HREC before they were utilised.

### Data analysis

We reviewed the data following a thematic approach using framework analysis, a matrix-based system for organising, reducing, and synthesising data [40]. In initial analysis, first line codes were generated from IDI and FGD guides by two coders. Codes were modified after reading through the transcripts. We drafted a codebook, shared among the team, who analysed and double-coded two to three transcripts in order to clarify and resolve any disagreements by consensus before a final codebook was developed. The codebook was then imported into NVivo 10 (QSR International, Melbourne, Australia) as nodes, which were used to extract text from the transcripts. The extracted chunks of text were compiled from all IDIs, FGDs and KIIs under specific codes and sub-codes. These thematically-organised chunks of data were then reviewed to explore the “detail and distinctions” [40] of each and synthesised into meaningful themes, such as ‘overall acceptability and willingness to use NPTs’. In order to analyse NPT preferences by population, coding and synthesis was first done for each participant group (i.e. adolescents) before codes and general themes were compared and contrasted across different participant groups. This process was also implemented to facilitate comparisons between trial-naïve and trial-experienced participants, when this information was available, although as noted above participants were often reluctant to identify themselves as trial-experienced due to HIV stigma.

An additional aspect of analysis emerged during the coding and synthesis process. After noting that participants expressed initial enthusiasm for NPTs but more hesitancy as they reflected on what using each NPT product would mean for them in practice, we also coded each transcript with this ‘evolution’ of acceptability in mind; initial acceptability tended to be enthusiastically expressed earlier in the transcripts with more nuanced and conditional acceptability expressed later in the transcripts.

## Results

From November 2013 to February 2014 we recruited 48 participants from key and vulnerable populations, and eight key informant healthcare workers. The majority of participants were 28 years-old and under, a group denoted in South Africa as high risk [41]. Table 1 profiles participant and key informant demographic characteristics, and data collection methods by population. We then present overarching themes and specific sub-themes that emerged from the data, along with exemplar quotations (see S5 Appendix for additional quotations).

**Table 1:** Overview of participant and key informant demographic characteristics and data collection methods by population.

Characteristics	Adolescents	Heterosexual Women	Heterosexual Men	MSM	KI Healthcare Workers
(N = 56)	(n = 14)	(n = 10)	(n = 9)	(n = 15)	(n = 8)
**Age, years**					
Range	15–17	18–32	19–37	20–51	21–50
Mean (SD)	15.64 (0.84)	23.20 (4.98)	25.00 (6.52)	27.87 (8.73)	36.71 (9.86)
**Gender**					
Male	8	-	9	15	4
Female	6	10	-	-	4
**NPT trial experience**					
No	13	6	4	7	-
Yes	1	4	5	8	8
**In-depth interviews**	4	2	2	4	8
**Focus group discussions**					
Number of groups	2	1	1	5	-
Number of participants per group	5, 5	8	7	5, 6	-

MSM, men who have sex with men; KI, key informant; NPT, new prevention technology

*Note*. Adolescents all self-identified as heterosexual. Key informants’ sexual identity was not elicited.

### Overall acceptability and willingness to use NPTs

Participants across populations expressed initial enthusiastic willingness to use NPTs, and key informant healthcare workers voiced their support for the potential of NPTs to enhance HIV prevention. In their subsequent discussions, however, both participants and key informants reflected on the implications these products might have on participants’ everyday lives, and began to consider product-related factors that they perceived as peculiar or that required a change in their lifestyle and everyday practices. As a result, all participants expressed various reservations about possible use of NPTs. Factors such as possible side effects, degree of efficacy, dosing regimen and duration of protection were key features influencing acceptability. In addition, varying degrees of discomfort that participants anticipated experiencing from the use of injectable products and a vaginal microbicide ring were noted as potential barriers to NPT acceptance. Conversely, participants’ conceptualisation of the products as ‘a familiar pill’ and therefore ‘easy to use’, or a ‘lubricant’ and therefore ‘easy to apply’ had a positive influence. In general, while consideration of specific product characteristics provoked a shift in participants’ overall acceptability, it also revealed product preferences that varied by population (see Table 2).

**Table 2:** Product acceptability and preferences by population.

Population	Product Preferences and Focal Attributes
Heterosexual women	Preferred vaginal ring (used monthly) and HIV vaccine (annually) due to their longer duration of protection, thus not requiring daily or event-driven use (e.g., before each sexual encounter)
Adolescents	An HIV vaccine was most preferred due to longer-term protection; female adolescents also indicated preferences for a vaginal ring
Heterosexual men	Preferred oral PrEP (pre-exposure prophylaxis) because thought it easy to use and familiar, but concerned about possible side effects
Men who have sex with men (MSM)	Preferred rectal microbicide, seen as easy to use, but concerned about dosing regimen (daily dose may be missed, event-driven use equated to condom use)
Key informant healthcare workers	Regarded various products as a possibility for their clients, but emphasised concerns about barriers to real-world uptake and effectiveness due to social-structural contexts that produce vulnerability

### Acceptability and willingness to use NPTs impacted by everyday experiences

Participants’ responses revealed the extent to which their willingness to use various NPTs is mediated by their present-day experiences in social and intimate relationships, and the broader social-structural context.

#### Inconsistent condom use

A dominant response from female participants conveyed the benefit of NPTs as counteracting the general ineffectiveness of condoms in their everyday lives. Their responses support some of the initial impetus for NPT development, which argues that NPTs will facilitate women’s autonomy. These include concerns about male partners’ not wanting to use condoms, their feigning condom use (“a person would say he has put it on and yet hasn’t” [Adolescent female, product-experienced, IDI]), and the challenges women face in condom negotiation. Analogously, we observed from heterosexual male responses that their acceptability of NPTs is largely mediated by practices relating to traditional masculine roles and behaviours in the social and sexual arena, where men have more control than women in sexual activity. Heterosexual male and MSM responses indicated not wanting to use condoms all the time: “we as guys when you do the second round you don’t want to see the condom and you want flesh” [Heterosexual male, product-naïve, FGD]. Gel-based topical microbicides were seen as providing protection to female and male sexual partners despite lack of condom use: “The importance of the gel, that you are protected so that even if you get to that point of having had enough of this thing [condom]…” [Heterosexual male, product-naïve, IDI]. These responses indicate the extent to which participants gauge acceptability of NPTs in light of their interactions and experiences in the context of existing gendered norms around sexual behaviour and condom use.

#### Unplanned and forced sexual encounters

Participants consistently described the use of NPTs as significantly beneficial in hypothetical instances of sexual assault. Both women and MSM envisaged the ever-present threat of rape as a pre-eminent risk for HIV infection: “things happen in South Africa and gays get raped every day” [MSM, product-naïve, IDI]. NPTs perceived as offering daylong (rectal microbicide) or month-long (vaginal ring) protection were seen as a safeguard against HIV in the event of forced sex: “…if she had the ring inserted and she’s going wherever, and she is grabbed by someone and that person has HIV; if that person rapes her, the chance of getting HIV is less” [Adolescent female, product-experienced, IDI].

MSM further reported on the usefulness of NPTs in maintaining spontaneity in cases of unplanned consensual sex, with statements such as “You meet a guy, there’s no time for a condom; if you have a microbicide (gel), you use it” [MSM, product-naïve, IDI], or references to weekend fun involving alcohol and drug use, and sex: “So I would have to use that gel on a weekend basis because you know how it is in the townships, we have fun on weekends and certain excitement and party” [MSM, product-experienced, IDI]. Women anticipated benefits of products that would not interrupt spontaneous encounters and invoke male partners’ concerns: “you had not prepared yourself that you would be doing this… ‘Please, just a minute, *Bhuti* (brother), I’d like to insert this thing…’ ‘What is it that you want to put in…for what?’ …when you have the ring, there is nothing he will ask you” [Heterosexual woman, product-naïve, FGD]. Thus, participants envisioned that NPTs would reduce the risks of acquiring HIV through spontaneous as well as forced sexual encounters.

#### Transmission in serodiscordant couples

NPTs, regardless of degree of efficacy, were described as an option for serodiscordant couples, including those who want to conceive a child without the risk of HIV transmission. A particular focus emerged on HIV-negative male partners of HIV-positive women. A male adolescent explained, “For example, I don’t have HIV and my partner is HIV-positive and perhaps I want to have sex with her; and therefore it’s imperative that I drink the pill because I don’t want HIV…[Adolescent male, product-naïve, FGD]. A healthcare provider described, in regard to conceiving a child, “there are also patients whereby the female is positive, the male is negative and they come to you…[and] they tell they want a baby” [Nurse, female, KII]. These reality-based concerns reflect a generalised epidemic in which HIV incidence is four times higher among females aged 15–24 compared with males of the same age [36].

#### Accidental exposures

There appeared to be a general belief that the risk of HIV transmission resulting from incidents such as motor vehicle collisions would be reduced if participants used NPTs, especially products with longer duration of protection: “It is not only for sexual intercourse according to my way of thinking, because you can be in an accident in a car with someone who is HIV-positive” [Heterosexual man, product-naïve, FGD]. Another participant echoed this concern, saying: “It’s better than if you use it every day, whatever microbicide you use, so that you are protected; because sometimes an accident can happen and there is a friction of blood, and you have a cut in this accident and then you get HIV, so it’s better that you use it monthly” [Adolescent female, product-naïve, FGD]. This added benefit supported willingness to use NPTs once available.

### Contexts of vulnerability as barriers to acceptability

#### Prioritising prevention

In the era of AIDS, more so amidst a generalised epidemic, the expectation is that individuals will view prevention as a key component of health and therefore show enthusiasm towards a new menu of prevention options. On the contrary, the data suggest that pervasive insecurity emanating from the social and economic contexts in which people live (i.e., poverty, overcrowded housing, unemployment) produces a risk environment permeated by alcohol and drug dependence, and depression, which may deemphasise HIV prevention. A community outreach worker represented in stark terms the priorities she observed among local communities: “The majority of people in this surrounding area, they talk about alcohol and money. So now if you are going to tell them that you need to insert the ring or apply the gel, they will tell you, ‘where will I find time to go apply the gel, while I just met a guy in the tavern…?’” [Community outreach worker, female, KII]. Another community outreach worker described the extent to which economic dependency creates vulnerability for women, in characterising her community’s expected responses to NPTs: “I did not know that I will go to the tavern [local bar] and there is this good looking guy, he is into me, he wants to buy me a drink; I can’t say no because I want that alcohol and I don’t want to miss the guy…” [Community outreach worker, female, KII].

Although the risk environment may be understood logically as *motivating* NPT acceptability and uptake, this environment also emerged as *constraining* individual choices, capabilities, and motivations to prioritise and implement HIV prevention measures in participants’ daily lives.

#### Women’s ability to make decisions

Accounts from adolescent and adult women participants are commensurate with gender relations narratives that describe an imbalance inextricably tied to power, male domination, and other forms of inequality that are responsible for the lack of women’s involvement in important life decisions. Exacerbated by their lack of education, women’s inability to challenge the status quo also presents serious implications for their health seeking behaviour. Key informants, in particular, rather than participants themselves, articulated education and empowerment of women as central to NPT uptake: “But I do think it is fundamentally about empowerment and one’s feeling of agency over one’s life, which may not necessarily be present in someone who has been downtrodden for their entire lives” [Policymaker, male, KII].

Key informants denoted the ambivalence and sometimes fear that women would experience if they were to forego partner consultation and covertly use NPTs. Despite full knowledge of their partners’ non-monogamy, women were described as being reluctant to use NPTs for fear of invoking mistrust in their relationship. Key informants depicted the private deliberations that women engage in as they consider using the products, often tied to social-structural factors such as their degree of economic dependence on their partners: “…you know you are using sex or you are allowing yourself to be put in risk conditions because you want to put food on the table for your kids” [Policymaker, female, KII]. These responses indicate the importance of considering how gendered relationship dynamics in the larger context of social and economic inequities may influence uptake of NPTs for women in both long-term and transitory relationships.

#### Healthcare system barriers

Overwhelmingly, participants expressed concerns regarding the “architecture of public clinics”. Despite viewing public clinics as possible places for NPT distribution, participants expressed reluctance towards utilising these clinics due to protracted waiting times and perceptions that clinic staff are judgmental and condescending: “I think it was in 2009, and since I had my family planning then I never had contraception again, because at that clinic they judge you and they would just undermine you and ask, ‘what are you doing family planning for being so young?’” [Heterosexual woman, product-experienced, FGD]. Participants suggested that it would be difficult to have individuals who are not ‘sick’ standing in queues for hours, and meeting with staff they consider unprofessional and rude, in order to obtain prevention products. Participants also anticipated HIV stigma as a barrier to clinic distribution of NPTs: “They will judge us…it will seem as if I have gone to get ARVs [antiretroviral drugs] and yet I had come for microbicides” [Heterosexual woman, product-naïve, FGD].

Key informants also described limitations in the healthcare system, although they emphasised challenges in the supply chain and other logistical barriers rather than HCP behaviours and attitudes. A HCP invoked the overall lack of resources in the public health system in anticipating insufficient supply of NPTs, but also foresaw challenges in handling a new caseload of patients seeking NPTs in addition to the existing caseload of persons living with HIV: “There won’t be enough stock. And how is the clinic going to take these people, like, the clinic has sick people…so where are they [NPT users] going to go through?” [Nurse, female, KII]. Although HCP did not directly address stigma as a barrier to NPT uptake, this scenario further supports participants’ reported concerns about HIV stigma in that in seeking out NPTs through the existing and overcrowded healthcare system, they will be misconstrued as people living with HIV who are seeking treatment.

#### Beliefs about traditional therapies

A few participants described beliefs in traditional healing practices that might conflict with NPT acceptability: “perhaps I’ve been diagnosed as having evil spirits…; if I drink the pill they can become cross and say that I’m mixing my culture with western culture….” [Adolescent male, product-naïve, FGD]. However, they also demonstrated accommodation of traditional beliefs in particular product preferences. In the following scenario, a participant contrasts a vaginal microbicide with a vaginal ring, construing that only the latter requires insertion of a ‘foreign’ object in one’s body: “Because the gel doesn’t require one to insert things, you just have to apply it or apply it to that private part, the ancestors wouldn’t complain; better than the others….” [Adolescent female, product-naïve, FGD]. Thus, people may oscillate between religious and cosmological frameworks, which may pose both challenges and opportunities for NPT acceptability and adherence in South Africa.

## Discussion

As new HIV prevention technologies transition from the laboratory to clinical trials, to dissemination and implementation, it is critical to understand acceptability and willingness to use such products among key and other vulnerable populations at high risk for HIV acquisition. The findings of this study reveal the extent to which social-structural factors may frame and circumscribe the acceptability of new prevention methods among important end-user populations in resource-constrained settings. This is observed in many participants’ initial and enthusiastic reactions regarding potential NPT uptake, until they fully conceptualised what it would mean to use the products in their daily lives.

Beyond particular product preferences, we identified that crucial to NPT acceptability and future implementation is how individuals integrate their conceptualisation of product-associated benefits with their everyday experiences [24,30,42]. Many individuals across vulnerable populations of adolescents, adult women and MSM described the utility of NPTs in terms of the perilousness of everyday life in informal communities in South Africa. Participants described how they see these products as attractive options in contexts where condom use is irregular or non-existent and difficult to enforce, sexual violence is commonplace, and accidental exposures may occur.

Importantly, not all participants reported being motivated to use NPTs due to perceptions of circumstances outside of their control, such as non-consensual sexual encounters or accidental HIV exposure. Some individuals were also motivated to use NPTs in highly intentional contexts, such as when a serodiscordant couple wishes to conceive a child, or in circumstances in which an individual foresees the possibility of consensual sex but might not consistently have access to or choose to use a condom. This reaffirms the motivations behind the development of NPTs, linking HIV transmission with vulnerability among populations such as MSM, adolescents, and adult women, as well as highlighting the need to differentiate among diverse user groups’ preferences and needs [43–46].

To that end, we identified different general preferences for NPTs by population in the contexts of participants’ everyday lives, highlighting the importance of both increasing available HIV prevention options and identifying population-specific concerns and needs. Both adolescent and adult women’s preferences for a vaginal ring and HIV vaccine were expressed in the context of, first, strong concerns about male (particularly married/steady) partners’ desires (often not to use a condom) and approval; second, anticipated negative reactions to women being perceived as mistrusting, asserting independence or otherwise thwarting male dominance; and, third, the ever-present context of rape. These gendered concerns emerged more prominently in the case of oral PrEP and were often cited as a rationale for women’s preferences for a vaginal ring and HIV vaccine—similar to preferences elicited from women in the VOICE (MTN-003D) trial of oral PrEP and topical gel use [47]. A qualitative study of women who participated in an open-label PrEP trial in Cape Town identified anticipated stigma, and resulting nondisclosure of study participation and PrEP use to male partners and friends, as a barrier to PrEP use in the trial [48]. Previous research on vaginal microbicides similarly indicated acceptability to be contingent on male partners, including concerns about impact on sexual pleasure and duration of protection, with differences across women in different settings [42,49–51]. A vaginal ring may obviate some of women’s reality-based concerns about the demands and risks of product usage that rendered topical vaginal microbicides inefficacious [18,21] and impeded PrEP use [48] in clinical trials; however, adherence remained a challenge in a Phase III vaginal ring trial (ASPIRE; MTN-020), particularly among younger women [5].

Adolescent males expressed preferences for an HIV vaccine. A vaccine was envisioned as providing long-term protection amidst HIV serodiscordant relationships and as convenient given the stigma, and the ongoing hassle and cost, of purchasing and using condoms. A previous study including young men in South Africa similarly noted participants’ concerns about HIV stigma as a factor in HIV vaccine acceptability, with motivations based on no longer having to use condoms [52]. Heterosexual male adults preferred PrEP given their stated familiarity with oral medications, antipathy towards vaccines and mistrust regarding injections. Concerns about HIV vaccine efficacy [53] and mistrust of HIV vaccines [54] have similarly been identified among U.S. adults, particularly ethnic minority populations. The apparently lesser mistrust of HIV vaccines among adolescent males compared to male adults in the present study may bode well for roll-out of future HIV vaccines in South Africa.

MSM reported overall preferences for a rectal microbicide in the context of familiarity with lubricant gels, unpredictability of the timing and place of sexual encounters, and the pervasive risk of rape. The latter is confirmed by a population-based study in South Africa, including the Eastern Cape, which identified a high prevalence (~10%) of male-on-male sexual violence and sevenfold higher rates of victimisation among MSM than other men [55]. High acceptability of rectal microbicides in development has been identified across diverse MSM, with acceptability influenced by specific product attributes and applicator properties, as well as sociocultural contexts [24,56–59].

Our findings of different overall product preferences by population supports the need for location-population-specific preparedness research in planning for successful NPT dissemination and implementation. However, the present findings also reinforce critical research linking the epidemiology of HIV and AIDS to the social-structural context in South Africa [15,16], which presents enduring barriers that may impact NPT implementation. Social and structural conditions of pervasive poverty, unemployment, inequities on the basis of race, gender and sexuality, HIV stigma, and an under-resourced healthcare system in these peri-urban communities emerged as contributing to local risk environments characterised by widespread alcohol and drug use, sexual violence, depression and fear that produce vulnerability to HIV infection [60]. The effectiveness of NPTs in controlling the epidemic in South Africa may be contingent on understanding and addressing the historical, social, political and economic contexts in which they are to be rolled out [15,52,61,62].

Overall, our findings support a conceptualisation of NPTs as complex and evolving technological solutions that require ongoing, complementary investigations of end-user populations’ preferences, priorities, and the social-structural contexts in which they live, in order to facilitate successful uptake and adherence. They further challenge conceptualisations of HIV prevention overly predicated on individual choice, in which an expanded ‘menu’ of product options resolves limitations on individual behaviour that, for example, constrain the effectiveness of male condoms. Although eliciting end-user choices and increasing NPT options is certainly constructive, our findings also emphasise that individual choices—particularly among populations most vulnerable to HIV acquisition—are made under sometimes severe and enduring social-structural constraints. However, rather than despair at the incompleteness of technological solutions, these challenges indicate that the introduction of NPTs presents renewed opportunities to integrate biomedical, behavioural and social-structural approaches in combination HIV prevention [63–65]. By exploring location-population-specific contexts of NPT acceptability, as in the present study, we may support the leveraging of opportunities particular to each product (e.g., a female-controlled vaginal ring that may reduce reliance on condom negotiation at every sexual encounter) using multifaceted and empirically-based strategies to support product preparedness, introduction and roll-out. These include tailored implementation approaches that address community perceptions and social norms (e.g., use of a vaginal ring as offending the spirits or as inviting HIV stigma), community priorities (e.g., not needing to interrupt sexual encounters through use of a vaginal ring that could be applied monthly), and community concerns (e.g., engaging men in acceptability of women’s use of a vaginal ring) based on formative social research conducted in context.

This study has several strengths and limitations. Participants were recruited from among the most vulnerable communities in the world, in resource-constrained South African townships amidst an enduring generalised HIV epidemic. These are precisely populations for whom NPTs are most sorely needed. Although we initially intended to contrast perceptions of product-naïve and product-experienced participants, this proved challenging due to HIV stigma and, by association, stigma around HIV clinical trial involvement: participants generally opted not to identify themselves as former or current trial enrolees and, as a result, some reportedly ‘trial-naïve’ participants may have had prior experience with these products. Our data did not suggest group differences by clinical trial involvement, but we are unable to make definitive statements about possible systematic differences in product acceptability between trial-naïve and trial-experienced individuals. For one, participants’ non-disclosure of trial involvement is instructive in demonstrating the tangible influence of stigma on HIV prevention trial involvement and for future NPT roll-out. Furthermore, product-naïve and product-experienced participants lived in the same high HIV prevalence communities, resource-limited settings that present pervasive social-structural barriers to NPT implementation. Our inclusion of former and current trial participants further supports the validity of our findings, as they are individuals who have direct experience with some of the prevention technologies assessed. Moreover, although PrEP is now approved for use in South Africa [66], it was not yet licensed at the time this study was conducted. Thus, while the other NPTs assessed are still in the development pipeline, the lack of access to PrEP through the public health system in South Africa may have rendered it as ‘hypothetical’ to participants as the other NPTs. Coupled with participants’ lack of initial awareness and knowledge of PrEP, similar to the other NPTs, this suggests that these differences did not influence product acceptability.

We also acknowledge the potential overlap or close correlation between certain population groups, such as older adolescents and younger adults, as well as lack of specific recruitment of same-sex identified adolescents, for whom we deemed the risks of being ‘outed’ in the process of obtaining parental consent unacceptable. As such, the differing preferences of participants across these vulnerable populations may not be as distinct as the data in this study may suggest; nevertheless, the findings demonstrate population-specific concerns and challenges that impact on NPT preferences and acceptability. Furthermore, while we identified trajectories of NPT acceptability that emerged within IDI and FGD transcripts, whereby participants’ initial highly enthusiastic acceptability was tempered as they delved into the realities of utilising NPTs in the context of their everyday lives, future longitudinal research using qualitative as well as quantitative methods may help to evaluate evidence for these trajectories. As in any qualitative investigation, the findings may not be generalisable to other peri-urban communities outside of Cape Town, although many of the social-structural challenges identified—such as low rates of formal education among women, and high rates of sexual violence perpetrated against women and MSM—are common among informal communities in South Africa and elsewhere. Finally, although we described products that represented the current state of emerging science in biomedical HIV prevention, NPT products and characteristics (e.g., injectable long-acting PrEP, in development [67]) as well as acceptability and preferences may shift when actual products become available.

## Conclusion

A new era of biomedical HIV prevention, including PrEP and other technologies in the prevention pipeline, is poised to make a substantial contribution to controlling complex epidemics around the world. To that end, it is crucial to develop strategies and policies to support NPT preparedness and implementation among populations at greatest risk for HIV infection, including those in resource-limited settings. The present findings, from among the most vulnerable populations globally, underscore the importance of combination HIV prevention strategies that take into consideration and address a) how individuals view and experience the products fitting into their everyday lives, b) enabling individuals to gain experience-based knowledge with NPTs, and c) the enduring social and economic contexts that produce and sustain vulnerability to HIV infection.

## Supporting information

S1 Appendix COREQ checklist. (PDF)

S2 Appendix In-depth interview and focus group discussion questions. (DOCX)

S3 Appendix Xhosa versions of in-depth interview and focus group discussion questions. (DOCX)

S4 Appendix Key informant interview questions. (DOCX)

S5 Appendix Themes, dimensions and exemplar quotations. (DOCX)
