# Supplementary material for: Contexts of vulnerability and the acceptability of new biomedical HIV prevention technologies among key populations in South Africa: A qualitative study
Source: PLoS One. 2018 Feb 8;13(2):e0191251. doi: 10.1371/journal.pone.0191251 (PMC5805172; doi:10.1371/journal.pone.0191251)
Supplement: S2 Appendix — (DOCX) [file pone.0191251.s002.docx]

**Contexts of Vulnerability and the Acceptability of New Biomedical HIV Prevention Technologies among Key Populations in South Africa: A Qualitative Study**

**S2 Appendix. In-depth Interview and Focus Group Discussion Questions**

| **1. Have you ever heard of this type of research being done in your area? If so, how?**  *Possible probing topics:*   - If so, what do you know about these types of prevention that we are testing?   Have you ever been asked to participate in a prevention study? If so, did you agree or disagree? Why or why not? |
| --- |
| **EXPLAIN EACH PRODUCT using attached script: MICROBICIDE, ORAL PREP, VACCINES ONE BY ONE AND ASK THE FOLLOWING QUESTIONS EACH TIME.** |
| **2. Can you imagine yourself ever using this product? Why or why not?**  *Possible probing topics:*   - If yes, can you describe for me a scenario in which you could imagine wanting to use one of these products?   **3. When would you want to use it?**  *Possible probes*   - What would be the most convenient way for you to use it? Every day, just before sex, just after sex, every few months…?  1. **For Microbicides only: What delivery method would you prefer? i.e. the gel applicator or a ring?**   **5. Where do you think something like this should be made available to people?**  *Possible probes*   - If you would want to use it, where would be most convenient or comfortable for you to go and get it? - What do you think would make something like this easier or harder to use?   **6. Do you think that people you know would consider using this product? Why or why not?**  *Possible probes*   - Is there anyone you can imagine who this product would be good for? - Is there anyone you can imagine who you think this product would be NOT be good for? Why? |
| 1. **Do you currently use any types of medicines regularly?**   *Possible probes*   - If so, where do you usually get your medicine? At a pharmacy, at the clinic, from a Sangoma, etc? - If so, what types of medicines do you use? I.e. Creams, vitamins, supplements, etc.   **8. What are some good or bad things about getting medicines from your local clinic?**  *Possible probe*   - How does this make you feel? |
| **PERCEIVED RISK OF HIV** |
| **9. Do you normally use protection during sex?**  *Possible probe*   - If so, what? How often would you say that you use protection?  1. **Would you say that you are concerned about possibly getting HIV from a partner?**   *Possible probes*   - If so, to what extent? - Why? Are there any other measures that you take to protect yourself? |
| **EXPLORING PARTIAL EFFICACY CONCEPT** |
| **11. How would you explain to a friend what we mean by "partial efficacy"?**  **12. How would you explain to someone what these products do?** |
| ***Finally*** |
| **13. Thinking about all of these products we talked about, which one (or combination of them) would you most prefer to use? Why?** |
