# Supplementary material for: Contexts of vulnerability and the acceptability of new biomedical HIV prevention technologies among key populations in South Africa: A qualitative study
Source: PLoS One. 2018 Feb 8;13(2):e0191251. doi: 10.1371/journal.pone.0191251 (PMC5805172; doi:10.1371/journal.pone.0191251)
Supplement: S3 Appendix — (DOCX) [file pone.0191251.s003.docx]

**Contexts of Vulnerability and the Acceptability of New Biomedical HIV Prevention Technologies among Key Populations in South Africa: A Qualitative Study**

**S3 Appendix. Xhosa Version of In-depth Interview and Focus Group Discussion Questions**

| **IMIBUZO YODLIWANO-NDLEBE NENGQWALASELA YENGXOXO YEQELA**  **(iqela elikhethiweyo labasebenzisi )** | |
| --- | --- |
| **UKWAZISA NGEMIQULU YEZITHINTELO: Qalisa ngo “Kwazisa Ngemiqulu Yezithintelo” umbhalo-ngqangi.** | |
| **1** | **Sekhe weva ngoluhlobo lophando olwenziwayo kwindawo yakho yokuhlala? Ukuba kunjalo, njani?** |
|  | *Uphando olunokwenziwa:* |
|  | Ukuba kunjalo, yintoni oyaziyo ngezindidi zokuthintela esizivavanyayo? |
|  | Sekhe wabuzwa ukuba uthathe inxaxheba kwisifundo sezithintelo? Ukuba kunjalo, wavuma okanye awuzange uvume? Ngokuba okanye Hayi ngokuba? |
| **CACISA IMVELISO NGANYE usebenzisa umbhalo-ngqangi obekiweyo: I-MICROBICIDE, I-PREP ESELWAYO, IZIGONYO NGANYE-NGANYE KWAYE UBUZE LEMIBUZO ILANDELAYO IXESHA NGALINYE** | |
| **2** | **Uyazibona wena usebenzisa lemveliso? Ngokuba okanye Hayi ngokuba?** |
|  | *Uphando olunokwenziwa:* |
|  | Ukuba ewe, undicacisela ngesehlo apho uthi uzibona ufuna ukusebenzisa enye yezimveliso? |
| **3** | **Ungafuna ukuyisebenzisa nini?** |
|  | *Uphando olunokwenziwa:* |
|  | Yeyiphi eyona ndlela enokukulungela ukuba uyisebenzise? Imihla ngemihla, phambi kokwabelana ngesondo, emva kokwabelana ngesondo, rhoqo ngenyanga ezimbalwa…? |
| **4** | ***Yeye-Microbicide zodwa:* Yeyiphi iindlela yokunikezela ongayifuna? i.e. i-jeli efakwayo okanye umsesane?** |
| **5** | **Ucinga ukuba into enje kufuneka abantu bayifumane phi?** |
|  | *Uphando olunokwenziwa:* |
|  | Ukuba ungafuna ukuyisebenzisa, kuphi apho kungakulungela okanye kungalula ukuba uye uyokuyifumana? |
|  | Ucinga ukuba yintoni enokwenza into enje kubelula okanye kubenzima ukuyisebenzisa? |
| **6** | **Ucinga ukuba abantu obaziyo bangacinga ukusebenzisa lemveliso? Ngokuba okanye Hayi ngokuba?** |
|  | *Uphando olunokwenziwa:* |
|  | Ukhona umntu onokumcinga onokuthi lemveliso imlungele? |
|  | Ukhona umntu onokumcinga, ocinga ukuba lemveliso ayinakumlungela? Ngokuba? |
| **7** | **Ngoku zikhona iindidi zamayeza ozisebenzisayo qho** |
|  | *Uphando olunokwenziwa:* |
|  | Ukuba kunjalo, uqhele ukuwafumana phi amayeza akho? Ekhemisti, ekliniki, kwigqirha, okanye ezinye? |
|  | Ukuba kunjalo, ngamayeza anjani owasebenzisayo? i.e. izithambiso, izakha-mzimba, izincedisi okanye ezinye. |
| **8** | **Zeziphi izinto ezilungileyo okanye ezimbi ngokulanda amayeza kwikliniki esekuhlaleni?** |
|  | *Uphando olunokwenziwa:* |
|  | Ikwenza uzive njani lento? |
| **INDLELA EKUQONDWA NGAYO UMNGCIPHEKO WE-HIV** | |
| **9** | **Ingaba uqhele ukusebenzisa isikhuseli xa usabelana ngesondo?** |
|  | *Uphando olunokwenziwa:* |
|  | Ukuba kunjalo, intoni? Ungathi usebenzisa isikhuseli kangakanani? |
| **10** | **Ungathi uyaxhalaba ngokuba ungafumana i-HIV kwiqabane?** |
|  | *Uphando olunokwenziwa:* |
|  | Ukuba kunjalo, kangakanani? |
|  | Ngokuba? Zikhona ezinye indlela othi uzithathe zokuzikhusela? |
| **UKUHLOLA INGCINGA YOKUSEBENZA NGOKUNGAGQIBELELANGA** | |
| **11** | **Ungacacisa njani kwisihlobo ukuba sithetha ntoni ngo “kusebenza ngokungagqibelelanga”?** |
| **12** | **Ungacacisa njani emntwini ukuba ezimveliso zenza ntoni?** |
|  | **Okokugqibela…** |
| **13** | **Ngokucinga ngazo zonke ezimveliso esithethe ngazo, yeyiphi (okanye indibanisela yazo) onokukhetha ukuyisebenzisa? Ngokuba?** |
