# Supplementary material for: Contexts of vulnerability and the acceptability of new biomedical HIV prevention technologies among key populations in South Africa: A qualitative study
Source: PLoS One. 2018 Feb 8;13(2):e0191251. doi: 10.1371/journal.pone.0191251 (PMC5805172; doi:10.1371/journal.pone.0191251)
Supplement: S4 Appendix — (DOCX) [file pone.0191251.s004.docx]

**Contexts of Vulnerability and the Acceptability of New Biomedical HIV Prevention Technologies among Key Populations in South Africa: A Qualitative Study**

**S4 Appendix. Key Informant Interview Questions**

| **1. Have you ever heard of this type of research being done in your area? If so, where?**  *Possible probing topics:*   - If so, what do you know about these types of prevention that we are testing?   **2. What is your opinion about this type of research?** |
| --- |
| **EXPLAIN EACH PRODUCT using attached script: MICROBICIDE, ORAL PREP, VACCINES ONE BY ONE AND ASK THE FOLLOWING QUESTIONS EACH TIME.** |
| **3. Can you imagine yours clients using any of these product? Why or why not?**  *Possible probing topics:*   - If yes, can you describe for me a scenario in which you could imagine someone wanting to use one of these products?   **4. Realistically, do you think that people in this community would consider using this product? Why or why not?**  *Possible probing topics:*   - Can you imagine someone you know who you think this would be good for? Who this would not be good for? Why? |
| **PRODUCT PROVISION AND PROMOTION** |
| **5. Where do you think something like this should be made available to people? At a primary care clinic; pharmacy; home-based delivery, etc?**  *Possible probing topics***:**   - If so, what? How often would you say that you use protection? - Would you say that you are concerned about possibly getting HIV from a partner? - If so, to what extent? - Why? Are there any other measures that you take to protect yourself? |
| **EXPLORING PARTIAL EFFICACY CONCEPT** |
| **6. How would you explain to a friend what we mean by "partial efficacy"?**  **7. How would you explain to someone what these products do?** |
| ***Finally*** |
| **8. Thinking about all of these products we talked about, which one (or combination of them) would your clients most prefer to use? Why?** |
