# Supplementary material for: Contexts of vulnerability and the acceptability of new biomedical HIV prevention technologies among key populations in South Africa: A qualitative study
Source: PLoS One. 2018 Feb 8;13(2):e0191251. doi: 10.1371/journal.pone.0191251 (PMC5805172; doi:10.1371/journal.pone.0191251)
Supplement: S5 Appendix — (DOCX) [file pone.0191251.s005.docx]

**Contexts of Vulnerability and the Acceptability of New Biomedical HIV Prevention Technologies among Key Populations in South Africa: A Qualitative Study**

**S5 Appendix. Themes, Dimensions and Exemplar Quotations**

| **Themes & Dimensions** | **Exemplar Quotes** |
| --- | --- |
| **Acceptability and willingness to use NPTs impacted by everyday experiences** | |
| ***Inconsistent condom use*** |  |
| *Men lie about use* | Yes, because sometimes condoms are not 100% protective. Sometimes the condom can tear, you see. Sometimes a person would say he has put it on and yet hasn’t done so and you not being able to see that [you not aware of it]. So these microbicides could be another way of helping us when we are having sex. It would mostly help the youth because the youth don’t want the condom…. (Adolescent female, product-experienced, IDI) |
| *Aversion* | Because of the point that they don’t want condoms… may be …I don’t know…maybe because they really don’t want to use condoms…. (Community outreach worker, female, KII) |
| *Inconvenience* | I think the gel... I think it should be used once and not be like the condom which when you have used it you must again put on another one; it’s better to use it once…. (Heterosexual man, product-naïve, FGD) |
| *Condom fatigue and HIV fear* | I have chosen the gel, I think they [women] could use it considering the fact that it assists with the thing that a person doesn’t get infected at all by these infectious diseases. Even when we discuss as men, although a person is not open because he doesn’t want to be known at all and it’s not things we usually talk about, but you will find out that during the discussion you will hear one saying, “hey, this thing [condom] one gets tired of it, and after a time not having used it I become anxious. (Heterosexual man, product-naïve, IDI) |
| ***Unplanned and forced sexual encounters*** | |
| *Rape (female adolescent)* | People get raped; a person is raped not knowing that she is going to be raped and yet if she had the ring inserted and she’s going wherever and she is grabbed by someone and that person has HIV, if that person rapes her, the chance of getting HIV is less. The one I would choose is the ring…. (Adolescent female, product-experienced, IDI) |
| *Rape (gay man)* | Because things happen in South Africa and gays get raped every day and you report a rape case. Because you might get raped if you have injected [microbicide gel] in the morning at least you are on the safer side…. (MSM, product-naïve, IDI) |
| *Spontaneity* | You meet a guy there’s no time for a condom. If you have microbicide [gel] you can use it…. (MSM, product-naïve, IDI ) |
| *Weekend fun* | …for me I would say because of the current moment I’m in, I would not say I’m in a relationship…so I would have to use that gel on a weekend basis because you know how it is, in the townships we have fun on weekends and certain excitement and party [laughs]…. (MSM, product-experienced, IDI) |
| ***Transmission in serodiscordant couples*** | |
| *HIV-positive female partner* | For example, I don’t have HIV and my partner is HIV+ and perhaps I want to have sex with her and therefore it’s imperative that I drink the pill because I don’t want HIV…. (Adolescent male, product-naïve, FGD) |
| *Pregnancy* | Because there are also patients whereby the female is positive, the male is negative, and they come to you…[and] they tell they want a baby…so you can use that [referring to NPT] with the female…. (Nurse, female, KII) |
| ***Accidental exposures*** |  |
| *Accidental cut* | It’s better than if you use it every day, whatever microbicide you use, so that you are protected because sometimes an accident can happen and there is a friction of blood and you have a cut in this accident and then you get HIV, so it’s better that you use it monthly” (Adolescent female, product-naïve, FGD) |
| *Motor vehicle collision* | Because it is not only for sexual intercourse according to my way of thinking, because you can be in an accident in a car with someone who is HIV+ and you have taken this pill…and his blood mixes with mine and I won’t be infected, so I would use it (Heterosexual man, product-naïve, FGD) |
| **Contexts of vulnerability as barriers to acceptability** | |
| ***Prioritising prevention*** |  |
| *Unemployment, poverty and alcoholism* | Because of the environment, like the area that we are working at, most of the people don’t work, some…they are just uneducated, some they just don’t care about their lives as long as they are gonna get alcohol and money it’s done. And you come across [these individuals] each and every day in that kind of a situation. The majority of people in this surrounding area they talk about alcohol and money. So… now if you are going to tell them that you need to insert the ring…or apply the gel …they will tell you…where will I find time to go apply the gel, while I just met a guy in the tavern…. (Community outreach worker, female, KII) |
| *Economic dependence* | I did not know that I will go to the tavern and there is this good looking guy, he is into me, he wants to buy me a drink, I can’t say no because I want that alcohol and I don’t want to miss the guy…. (Community outreach worker, female, KII) |
| ***Women’s ability to make decisions*** | |
| *Illiteracy and women’s empowerment* | I really need to emphasise, when we actually need to promote this biomedical [prevention technologies], we must not leave out something that is very much crucial, which is women’s empowerment. So if you don’t empower women, you don’t actually make her knowledgeable and make known of these factors…it actually has to go hand in hand with that. In our country majority of the people are not literate. They actually are ignorant about things like this. So we really need to balance it…. (Policymaker, female, KII) |
| *Social mobilisation and empowerment* | Compliance is a problem that’s why I say it has to do with the readiness of an individual that you know before you actually market this. There has been a lot of social mobilization that people are empowered, they are educated, they are aware…. (Community outreach worker, female, KII) |
| *Economic dependence on men* | [The woman knows] I need to insert the ring. But are those men really receptive of whatever these women are going to be doing in order to protect and prevent themselves [from getting HIV]?…and addressing poverty is another thing. (Policymaker, male, KII) |
| ***Healthcare system barriers*** | |
| *Judgmental staff* | Yes, because even when I was participating in the study here, the last time I had family planning there was a study…I think it was in 2009 and since I had my family planning then I never had contraception again because at that clinic they judge you and they would just undermine you and ask, “what are you doing family planning for being so young?” The nurse is not ashamed of asking you something like that, saying too, “oh, no are you sleeping with boys already as young as you are”— they say hurting things…. (Heterosexual woman, product-experienced, FGD) |
| *Long waiting times and overburdened staff* | …at the [government] clinic I come in and get to the nurse and I fetch my folder and [because] at the clinic we are a lot [of people], it takes time and you have to wait for a long time; and when you get to the nurse and say you have come to get this, she would say, “Oh no this thing of yours is boring, and why do you have to have sex with a lot of men,” things like that, so there are a lot of challenges. (Heterosexual woman, product-naïve, FGD) |
| *Judgment and blame* | The nurses there take you to be a slut, they take you to be someone who has sex with everybody and you therefore don’t want to get AIDS, but because you are someone who doesn’t care you don’t want to use the condom. (Heterosexual woman, product-naïve, FGD) |
| ***Beliefs about traditional therapies*** | |
| *Traditional beliefs* | Because the gel doesn’t require one to insert things, you just have to apply it or apply it to that private part, the ancestors wouldn’t complain, better than the others…. (Adolescent female, product-naïve, FGD) |
| *Mixing cultures* | Perhaps I’ve been diagnosed as having evil spirits and I have been given a certain medicine to drink, and I use this medicine because I also want to be safe; if I can drink the pill they can become cross and say that I’m mixing my culture with western culture…. (Adolescent male, product-naïve, FGD) |

NPTs, New prevention technologies; IDI, in-depth interview; KII, key informant interview; FGD, focus group discussion; MSM, men who have sex with men
